# PhenoStras2022: A 2022 smartphone-based image and field phenology dataset for monitoring urban trees in Strasbourg, France

**DOI:** 10.1016/j.dib.2026.112502

**Published:** 2026-01-22

**Authors:** Clément Bressant, Pierre-Alexis Herrault, Anne Puissant

**Affiliations:** a LIVE UMR 7362 CNRS, University of Strasbourg, Strasbourg, France; b UAR 2013 CNRS, Data Terra/THEIA, Paris, France

**Keywords:** In situ, Digital hemispherical photography, Front-view photography, BBCH scale, Low-cost

## Abstract

Urban trees provide critical ecosystem services but remain vulnerable to climate change and urban environmental stresses. To improve understanding of their phenological dynamics and support reproducible urban vegetation monitoring, PhenoStras2022 is introduced, a dataset based on non-destructive, low-cost (using a portable smartphone-based setup) ground-based observations. Data were collected throughout a complete phenological cycle in 2022 across 40 green sites in Strasbourg, France. Two types of photographic acquisitions were conducted: Digital Hemispherical Photography (DHP) and Front-View Photography (FVP), complemented by field-based phenological observations following a simplified BBCH scale. The dataset is organized to enable a range of applications, including remote sensing products validation and urban climate studies. PhenoStras2022 addresses the current lack of ground-based urban phenology data and provides a robust foundation for analysing seasonal patterns and the resilience of urban trees. It also promotes participatory science by offering an accessible, replicable acquisition protocol, contributing to the strengthening of phenological monitoring networks in the context of a changing climate.

Specifications Table

**Table:** 

Subject	Earth & Environmental Sciences
Specific subject area	Phenology monitoring, Image processing
Type of data	Image (JPG), Table (CSV), GeoPackage (GPKG) / Raw and Processed
Data collection	The dataset was collected throughout 2022 to monitor urban trees across 40 green sites located within the urban area of Strasbourg (France) using a smartphone camera (Samsung SM-A528B/DS – 64 MP). It consists of two types of photographic acquisitions of the trees: Digital Hemispherical Photographs (DHP), captured from beneath the canopy using a removable 180° fisheye lens to provide an upward-looking view; and Front-View Photographs (FVP), taken with the smartphone's standard sensor for a direct, horizontal perspective of the crown. These image-based observations were complemented by a site-level phenological monitoring table using a simplified BBCH scale, providing field-based annotations of key developmental stages of the urban trees across the entire season.
Data source location	40 sites within the Eurométropole de Strasbourg, Grand-Est, France (48.57-48.62°N, 7.72-7.80°E)
Data accessibility	Repository name: EaSyData Data identification number: 10.57932/853b63f5-7816-481e-8e12-7b1f2f7f08e2 Direct URL to data: https://doi.org/10.57932/853b63f5-7816-481e-8e12-7b1f2f7f08e2
Related research article	C. Bressant, P. -A. Herrault and A. Puissant, Fine-Scale Phenology of Urban Trees From Satellite Image Time Series: Toward a Comprehensive Analysis of Influencing Factors, in IEEE Journal of Selected Topics in Applied Earth Observations and Remote Sensing, vol. 17, pp. 11685-11706, 2024, https://doi.org/10.1109/JSTARS.2024.3411304.

## 1 Value of the Data

• The dataset provides a unique collection of ground-based photographs acquired in an urban context, including both hemispherical and front-view images, using a low-cost and portable smartphone-based setup. It addresses the lack of accessible and well-documented reference datasets for urban phenological monitoring under operational and regulatory constraints.
• The availability of raw, high-frequency image data enables the development, testing, and benchmarking of image-processing workflows for phenological index extraction (e.g., Green Chromatic Coordinate (GCC) and Leaf Area Index (LAI) proxies), supporting the evaluation of relationships between canopy structural metrics and colour-based phenological indicators.
• It can be used to calibrate and validate satellite- and aerial-based phenological products (e.g., Sentinel-2, PlanetScope, drone imagery) at the local scale, contributing to multi-scale urban vegetation and climate studies.
• Owing to its reproducible acquisition protocol, low technical requirements, and availability of raw image data, the dataset provides a ready-to-use reference for training and testing methods before conducting field campaigns. It is also well suited for participatory science initiatives, teaching activities, and the harmonisation of urban phenology observations across sites and cities.

## 2 Background

Urban trees are essential components of urban ecosystems, providing a wide range of ecosystem services, including microclimate regulation [1], air quality improvement [2], stormwater mitigation [3], and habitat support for urban biodiversity [4]. They also play a key role in enhancing the physical and mental well-being of urban residents [5,6]. In the face of accelerating climate change, the ecological and social functions of urban trees are increasingly recognised as essential for strengthening urban resilience [7]. However, these trees are simultaneously exposed to intensified environmental stressors, such as rising temperatures, drought, and pollution, which can threaten their health and longevity [8]. Understanding the growth responses and phenological dynamics of urban trees under these pressures is critical for informing sustainable urban planning and green infrastructure management [9,10]. Yet, ground-based phenological data remain scarce, particularly in urban settings. Continuous monitoring of tree phenology across the year provides valuable environmental indicators that help assess tree condition, detect early signs of stress, and anticipate future trends in urban vegetation dynamics.

## 3 Data Description

The dataset was developed following a thorough review of existing methods, prioritising non-destructive, ground-based, and easily replicable techniques adapted to the constraints of urban environments. To overcome technical, regulatory, and logistical challenges, portable and affordable devices (specifically smartphones equipped with removable fisheye lenses) were used to collect high-frequency and accurate measurements. The dataset consists of two types of photographic acquisitions: Digital Hemispherical Photographs (DHP) and Front-View Photographs (FVP). The DHP images were captured using a removable 180° fisheye lens attached to the smartphone, enabling hemispherical canopy analysis. The FVP images were taken with the smartphone’s standard front-facing sensor to document visible foliage and canopy structure.

These complementary image acquisitions were designed to support the estimation of two key biological indicators related to urban tree phenology: Leaf Area Index (LAI), which reflects leaf quantity (leaf development), and Green Chromatic Coordinate (GCC), often used as a proxy for leaf vigor (leaf color changes). The dataset is accompanied by field-based phenological observations using a simplified BBCH scale, providing ground truth data for validation and phenological stage identification.

**Fig. 1:**
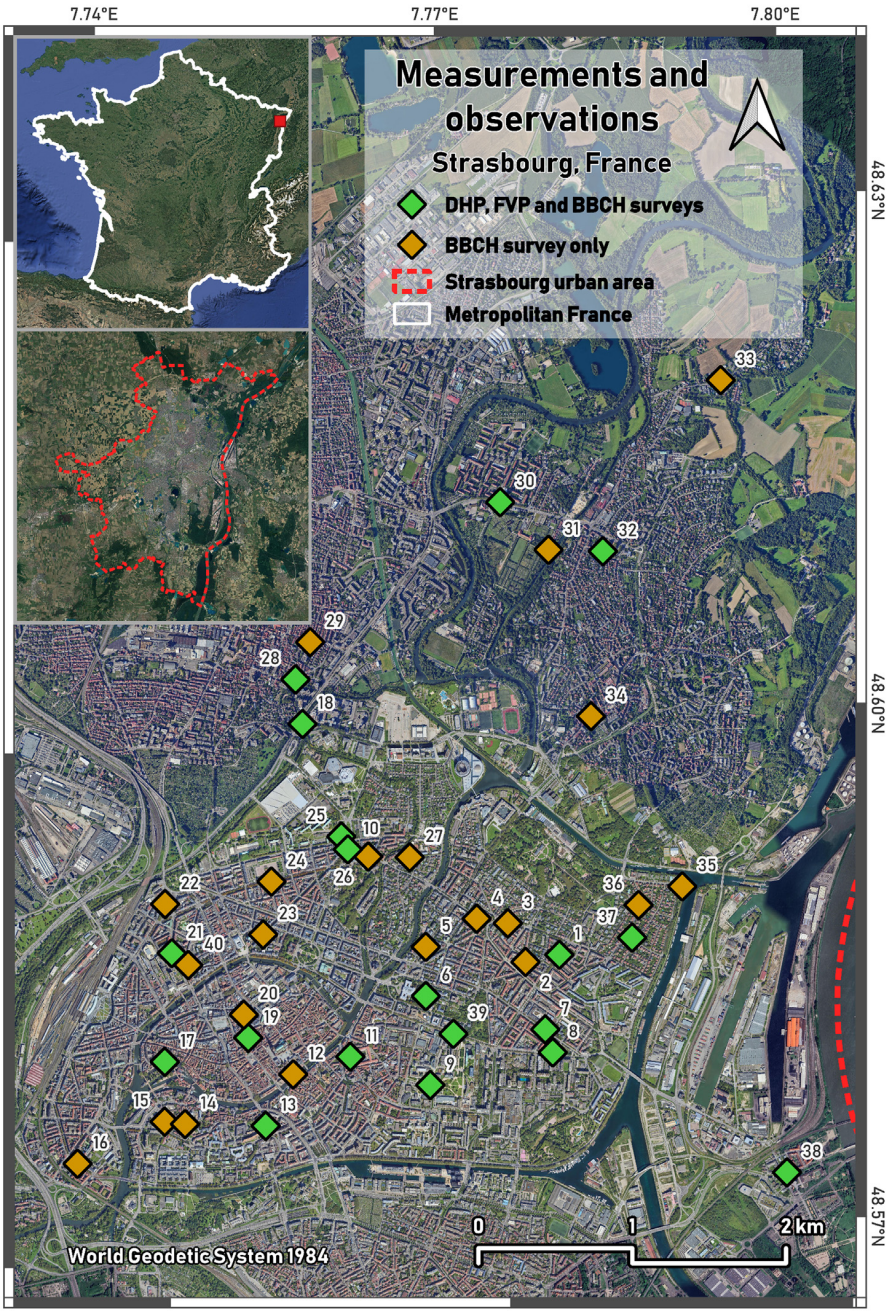
Location of green sites where all measurements and observations were conducted within the Strasbourg urban area. In green, all measurements were performed (DHP, FVP and BBCH), while in orange, only BBCH observations were carried out.

The PhenoStras2022 dataset includes a GeoPackage file containing the locations of the 40 study sites (polygons delineating the measured trees), along with their unique IDs, the tree species (Latin binomial names), and the type of measurements conducted (DHP + FVP + BBCH for 19 sites, and BBCH only for the remaining 21 sites). A location map is also provided in Fig. 1. The folder structures and filename formats of DHP, FVP and BBCH photographs are summarised in Table 1.

**Table 1:** Folder structure and filename format by data type (DHP, FVP and BBCH).

**Data Type**	**Folder Structure**	**Filename Format**
**DHP**	DHP/Week_<week>/Site_<site>/P<point>/	Week_<week>_Site_<site>_P<point>_<YYYYMMDD>_ <HHMMSS>.jpg Example: Week_16_Site_1_P1_20220420_111814.jpg *Note: the suffix _C indicates acquisition between trees.*
**FVP**	FVP/Site_<site>/	Week_<week>_Site_<site>_<YYYYMMDD>_aligned.jpg Example: Week_16_Site_1_20220420_aligned.jpg
**BBCH**	BBCH/Week_<week>/Site_<site>/	Week_<week>_Site_<site>_<YYYYMMDD>_<index>.jpg Example: Week_16_Site_1_20220420_1.jpg *Contents also include:* *- BBCH observation table (.csv)* *Note 1: Week 60 is absent (dormancy).* *Note 2: Week 14 was additionally included*.

For each image, the acquisition date and time are available either in the filename and/or in the metadata, while precise GPS coordinates and acquisition settings are systematically recorded in the metadata.

## 4 Experimental Design, Materials and Methods

### 4.1 Requirements for data collection

Prior to the field campaign, 40 deciduous tree sites, referred to as “green sites” (see Bressant et al. [11]), were selected within the urban area of Strasbourg, France. Site selection was informed by descriptive metadata from the OpenDataStrasbourg repository *Patrimoine arboré 2022*, ensuring consistency across sampling locations. In particular, selected sites featured homogeneous tree species and similar maintenance regimes to reduce variability due to external management factors.

**Fig. 2:**
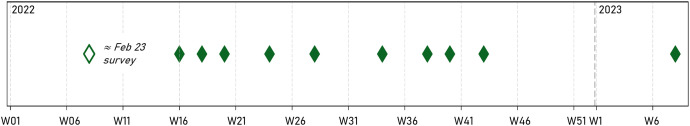
Approximate weeks of field surveys, with a total of 10 sessions. The final survey conducted in February 2023 (labelled as Week 60 in the dataset) corresponds to calendar Week 08 of 2023 and can be used as a proxy for Week 08 of 2022 for calibration purpose

Data collection was carried out from March 2022 to February 2023, covering a full phenological cycle of deciduous trees. To capture key seasonal transitions, measurement frequency was increased during periods of rapid phenological change, such as spring leaf-out (April–June) and autumn senescence (September–November). During these peak periods, sites were monitored approximately every 10 to 15 days, totaling 10 acquisition sessions over the year (see Fig. 2). The final data collection session in February 2023 was designed to mirror pre-leaf-out conditions equivalent to February 2022. This time point serves as reference or baseline for calibrating leaf-related indicators by capturing the canopy in a leafless state, with only woody structures visible.

**Fig. 3:**
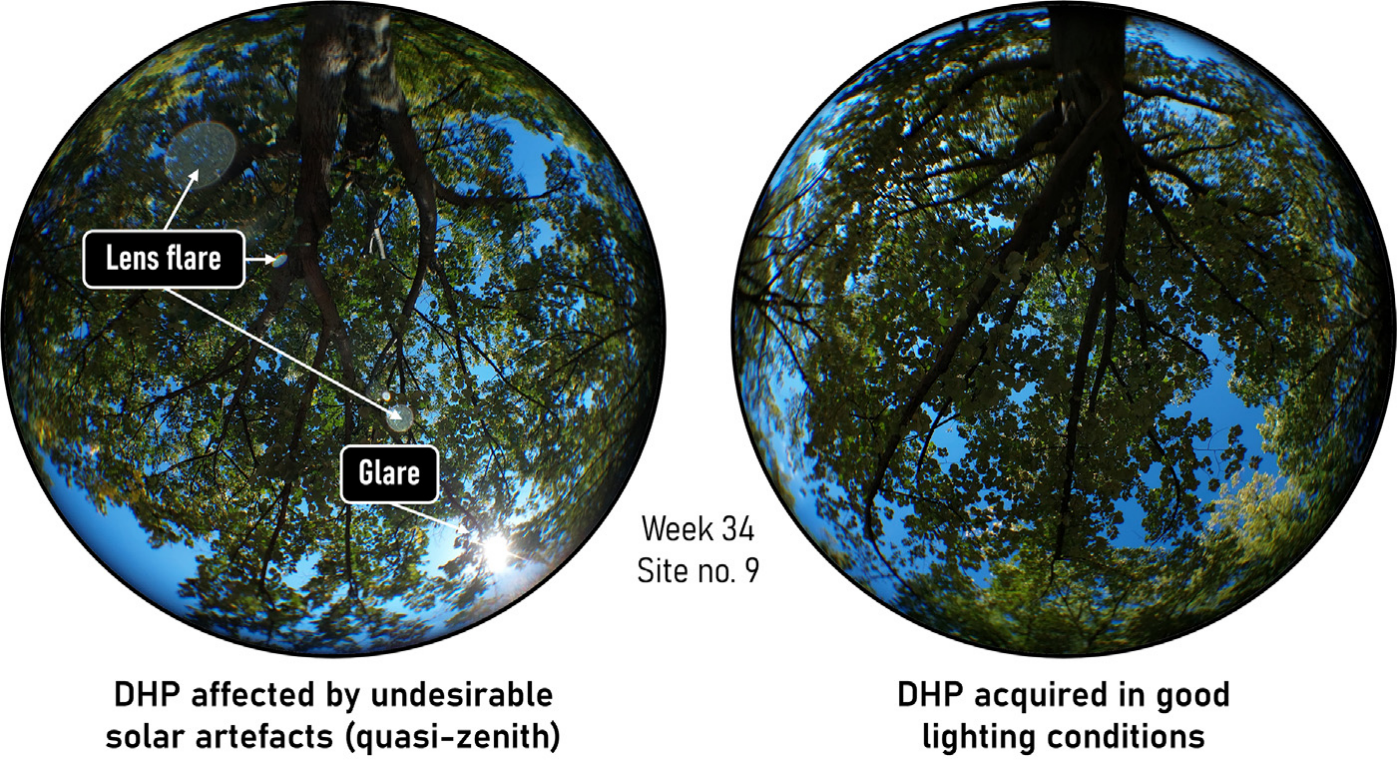
Example of the impact of lighting conditions on two digital hemispherical photographs. On the left, a photograph disrupted by the presence of artifacts; on the right, an optimal photograph without biases for subsequent calculations. Acquired on site no. 9 on August 23, 2022.

The integrity of subsequent index calculations (see next sections) was ensured by acquiring all photographs during daylight hours (approximately 7:30 am to 2:30 pm GMT), under favourable weather conditions. Dry weather and low wind were essential to prevent moisture from altering leaf orientation and to avoid motion blur due to foliage movement [12]. Acquisition consistency and image quality were improved by conducting measurements outside of solar zenith hours: direct sunlight and harsh shadows were minimised by favouring diffuse light conditions (e.g., blue skies or uniform cloud cover), which reduce overexposure, glare, and lens flare [13]. This issue is particularly relevant in digital hemispherical photographs when leaf cover is sparse, especially during transitional phenological stages, as incident sunlight is not filtered by a dense canopy (unlike in forest environments) [14]. The effects of direct light and shadow patterns also vary with the sun’s orientation and the cardinal direction of the sensor during acquisition [15]. An example illustrating appropriate light conditions during acquisition is shown in Fig. 3.

These challenges were mitigated and reproducibility was enhanced by conducting multiple DHP acquisitions around the same tree during each measurement session and, where possible, across several trees at the same site. Consistent lighting and weather conditions between survey dates were also prioritised to support comparability across the time series. The smartphone camera used and configuration (Samsung SM-A528B/DS - 64MP) were selected based on practical field testing and a literature review, with the aim of maximising contrast between tree structures (branches, leaves) and background elements (sky, buildings).

More precisely, the camera settings, including ISO sensitivity, shutter speed, aperture, white balance, autofocus mode, and no optical zoom, were optimised to meet the requirements of both hemispherical and front-view photography. These settings are detailed in Table 2. For simplicity and consistency, the same camera configuration was applied to FVP. Preliminary tests confirmed that this did not affect the accuracy of color-based processing tasks, such as vegetation index extraction.

**Table 2:** Camera settings for in situ data acquisition (Samsung SM-A528B/DS - 64MP), from Bressant et al., 2024.

	Value	Purpose	Objective
**ISO**	100	Light sensitivity	Low, to avoid the sensor being too sensitive to light, limit noise such as lens flare and poor pixel characterisation at the tree/sky interface
**Shutter speed**	1/3000s	Exposure time	Fast, to avoid the sensor receiving light for too long, and to more accurately capturing leaves (canopy movement can be significant due to wind, especially in urban corridors)
**Aperture**	f/1.8	Size of lens opening	Wide, to allow a significant amount of light, useful when canopies are dense and low (a common case in cities). Also limited by sensor capabilities
**White balance**	5000K	Color adjustment	Neutral, to correspond to the daylight colour/shade (particularly useful for next front-view digital photographs)
**Focus**	Autofocus	Sharpness	To enable clear shots to be taken at each of the sites (different tree crown heights)

### 4.2 Digital hemispherical photography (DHP)

Digital Hemispherical Photography (DHP) is a discrete upward-facing image acquisition technique that offers a non-destructive and repeatable method for assessing canopy structure, and particularly gap fraction, under minimal technical constraints [[16], [17]–18]. While DHP yields robust structural data, its accuracy is sensitive to environmental conditions and operator variability, making standardised acquisition protocols essential. For this dataset, a removable 180° fisheye lens (CVL-220 Somikon) was mounted on the smartphone to capture hemispherical images. At each green site, DHP images were taken around the target tree from the four cardinal directions (N, E, S, W). In cases where physical obstacles (e.g., fences or walls) limited access, three acquisition points were used. Additional hemispherical images were collected between trees in groves or linear alignments, spaced at regular intervals, to ensure spatial coverage at the site level.

**Fig. 4:**
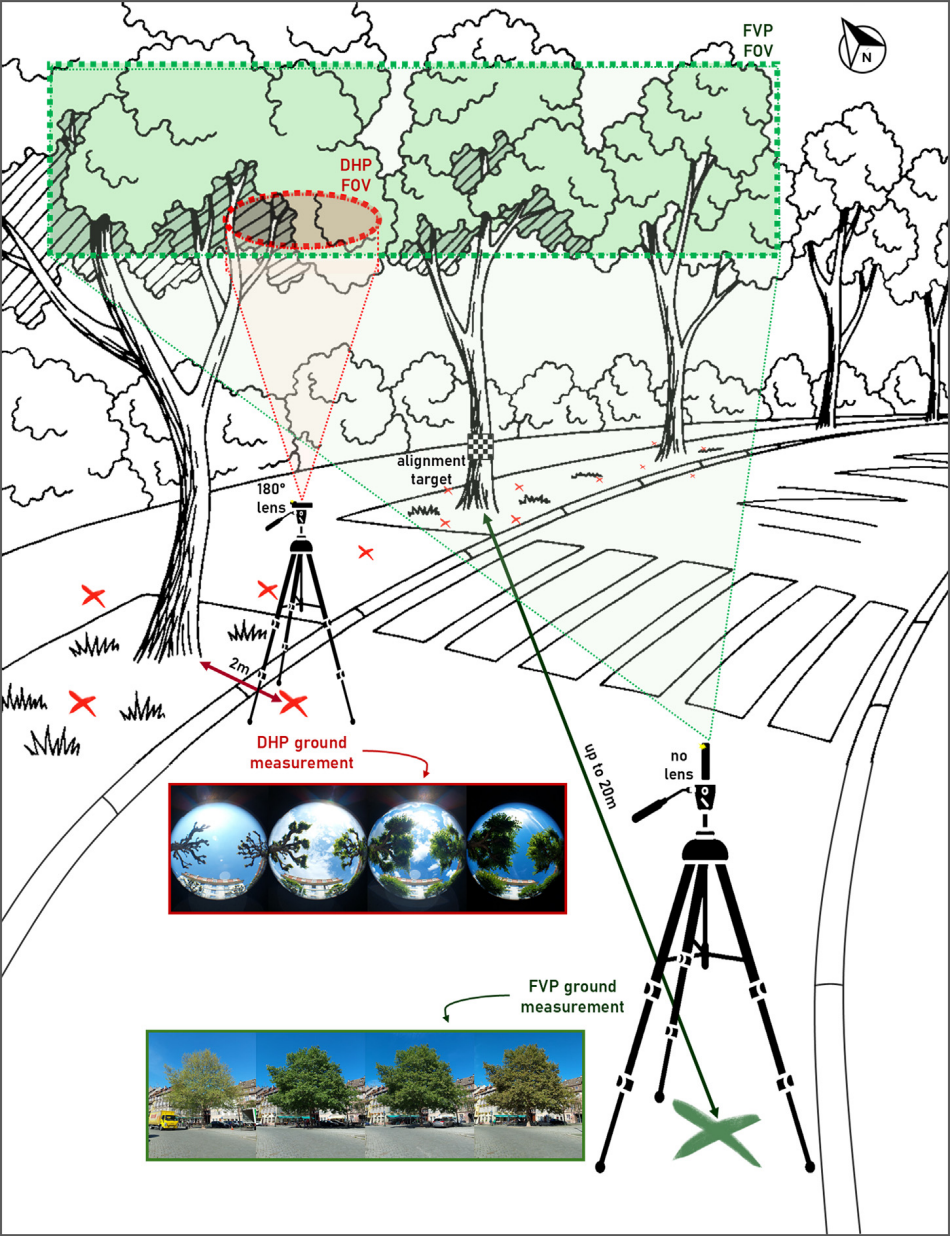
Immersive schematic view of field acquisitions (FVP and DHP). Examples for four dates are provided for each measurement (site no. 8 for DHP and site no. 13 for FVP). The depicted fields of view are intentionally reduced to highlight the areas of importance for the captures.

The smartphone was mounted on a tripod at a fixed height of about 1 metre and positioned 2 metres from the tree trunk, with the camera always facing the trunk to maintain measurement consistency. The tripod placement was marked on the ground, enabling the same acquisition geometry to be replicated across all survey sessions. This setup also ensured that any obstructions (e.g., signs, low-hanging branches) remained in the same position relative to the sensor over time, preserving temporal consistency in the dataset. Depending on the site configuration, the number of photographs per green site ranged from 4 (for isolated trees) to 15 (for clustered trees or groves). A schematic representation of the DHP acquisition setup is shown in Fig. 4, providing a visual overview of the protocol used.

### 4.3 Front-view photography (FVP)

Front-View Photography (FVP) consists of single-frame images capturing the upper portion of the tree, from the trunk to the canopy top, acquired during each field survey. Images were recorded at a resolution of 3468 × 4624 pixels using the smartphone’s standard sensor, with no additional lenses attached. To minimise glare and overexposure, photographs were consistently oriented northward, positioning the sun behind the camera and reducing shadowing on the canopy surface [19].

Each photograph was taken with the smartphone mounted on a tripod at a fixed height. The camera was aligned with a pre-positioned target on one of the selected trees at each green site, typically located 15 to 20 metres away, depending on tree height and local urban constraints. Acquisition points were georeferenced and remained fixed throughout the monitoring period for spatial and temporal consistency across all survey sessions. The schematic representation of the FVP acquisition protocol is also provided in Fig. 4.

To ensure consistent framing across all surveys, FVP also post-processed to correct for minor alignment offsets between acquisition sessions. The Auto-Align Layers function in ©Adobe Photoshop CS6 was used to automatically align the images from each site. This tool analyses structural features in the source image and applies either a Perspective or Cylindrical transformation, selecting the layout that yields the most accurate composite. This post-processing step further enhances temporal consistency and improves the reliability of downstream colour-based analyses, such as vegetation index extraction.

### 4.4 BBCH scale observations

The third component of this data collection involves visual observations of tree organic development, with a specific focus on active crown organs such as buds, stems, and leaves. These observations complement the photographic dataset by providing ground-truth information on key phenological transitions, thereby enhancing the interpretability of image-derived indices. To structure these observations, a simplified phenological scale was developed based on the BBCH scale (Biologische Bundesanstalt, Bundessortenamt und Chemische Industrie) for woody plants [20]. The simplification was motivated by two primary factors:

• Limited visibility of precise phenological stages in urban settings, due to pruning practices, ongoing maintenance, and various environmental disturbances;
• Species-specific gaps in phenological development, such as ornamental cultivars that flower without producing fruit, often due to genetic selection.

This adapted BBCH table still allows for a practical, scalable classification of phenological stages in urban trees while remaining compatible with standardised frameworks used in ecological and remote sensing research. The resulting simplified phenological coding system is presented in Table 3, and was applied consistently across the 40 monitored green sites throughout the 2022 observation cycle, as provided in the dataset.

**Table 3:** Simplified BBCH scale stages used in field surveys, with description.

BBCH stage	Description
**1 - Budburst**	From bud dormancy to bud burst
**2 - First leaves** (start)	0-50% of the first leaves have emerged
**3 - First leaves** (end)	50-100% of the first leaves have emerged
**4 - Stem / sprout** (start)	0-50% of sprouts / stems have reached their final size
**5 - Stem / sprout** (end)	50-100% of sprouts / stems reached their final size
**6 - Flowering**	From foral bud swelling to complete anthesis
**7 - Fructification** (start)	0-100% of the fruits have reached their maximum size
**8 - Fructification** (end)	0-100% of the fruits are mature
**9 - Fading / leaf fall** (start)	0-50% of leaves have changed colour or fallen off
**10 - Fading / leaf fall** (end)	50-100% of leaves have changed colour or fallen off

## Limitations

While the dataset was collected using a standardised and reproducible protocol, several limitations should be considered, as mentioned above:

• Urban constraints (e.g., buildings, street furniture, traffic, and human activity) occasionally limited the number and positioning of acquisition points, particularly for DHP. In some cases, only three directions (instead of four cardinal points) could be used due to physical obstructions.
• Lighting variability, despite being carefully managed, could not be entirely eliminated. Although efforts were made to ensure consistent acquisition during diffuse daylight conditions, some variation in light exposure, sky conditions, or surrounding reflectance may influence image-based index values (e.g., GCC).
• Smartphone camera limitations include a fixed aperture and limited manual control over certain advanced settings compared to professional equipment. However, extensive field testing and parameter tuning helped mitigate most of these constraints.
• Phenological stage visibility was sometimes affected by urban tree management practices such as pruning, which may alter crown structure or remove organs necessary for specific BBCH stages. In addition, not all developmental phases are observable across all species (e.g., absence of fruiting in sterile cultivars), which can reduce temporal continuity in BBCH data.

Despite these limitations, the dataset offers a high level of temporal consistency, methodological transparency, and practical relevance for applications in urban phenology, remote sensing calibration, and ecological monitoring.

## Ethics Statement

This dataset does not involve human subjects, animal experiments, or sensitive data.

## Credit Author Statement

**Clément Bressant**: Conceptualization, Methodology, Formal analysis, Software, Investigation, Data curation, Writing – original draft, Writing – review & editing.

**Pierre-Alexis Herrault**: Methodology, Software, Supervision, Validation.

**Anne Puissant**: Methodology, Supervision, Writing - Review & Editing, Funding acquisition.

## Data Availability

EaSyDataPhenoStras2022 (Original data)
